# Pronounced Phenotypic Changes in Transgenic Tobacco Plants Overexpressing Sucrose Synthase May Reveal a Novel Sugar Signaling Pathway

**DOI:** 10.3389/fpls.2015.01216

**Published:** 2016-01-11

**Authors:** Quynh Anh Nguyen, Sheng Luan, Seung G. Wi, Hanhong Bae, Dae-Seok Lee, Hyeun-Jong Bae

**Affiliations:** ^1^Department of Bioenergy Science and Technology, Chonnam National UniversityGwangju, South Korea; ^2^Department of Plant and Microbial Biology, University of California BerkeleyBerkeley, CA, USA; ^3^Bio-Energy Research Center, Chonnam National UniversityGwangju, South Korea; ^4^School of Biotechnology, Yeungnam UniversityGyeongsan, South Korea

**Keywords:** sucrose synthase, sucrose-degrading SuSy activity, endogenous sucrose, enhanced photosynthesis, shoot apical meristems, *WUSCHELL*, CycD cyclin, sucrose signaling pathway

## Abstract

Soluble sugars not only serve as nutrients, but also act as signals for plant growth and development, but how sugar signals are perceived and translated into physiological responses in plants remains unclear. We manipulated sugar levels in transgenic plants by overexpressing sucrose synthase (SuSy), which is a key enzyme believed to have reversible sucrose synthesis and sucrose degradation functions. The ectopically expressed SuSy protein exhibited sucrose-degrading activity, which may change the flux of sucrose demand from photosynthetic to non-photosynthetic cells, and trigger an unknown sucrose signaling pathway that lead to increased sucrose content in the transgenic plants. An experiment on the transition from heterotrophic to autotrophic growth demonstrated the existence of a novel sucrose signaling pathway, which stimulated photosynthesis, and enhanced photosynthetic synthesis of sucrose, which was the direct cause or the sucrose increase. In addition, a light/dark time treatment experiment, using different day length ranges for photosynthesis/respiration showed the carbohydrate pattern within a 24-h day and consolidated the role of sucrose signaling pathway as a way to maintain sucrose demand, and indicated the relationships between increased sucrose and upregulation of genes controlling development of the shoot apical meristem (SAM). As a result, transgenic plants featured a higher biomass and a shorter time required to switch to reproduction compared to those of control plants, indicating altered phylotaxis and more rapid advancement of developmental stages in the transgenic plants.

## Introduction

The transition from heterotrophic to autotrophic growth is one of the most important processes during the plant life cycle, as plants survive and develop independently from the quantities of carbohydrate and nutrients that accumulate in seeds (Koornneef et al., 2002; Finch-Savage and Leubner-Metzger, 2006). Light-induced biomass production during autotrophic growth occurs through chlorophyll in chloroplasts to produce photosynthetically fixed carbon compounds (such as triose phosphates—TP), which are later released into the cytosol (Bédard and Jarvis, 2005; Philippar et al., 2007). Sucrose is a primary sugar synthesized mainly from TP through the catalytic action of sucrose-phosphate synthase (SPS) and sucrose-phosphatase (SPP) in the cytosol. As a disaccharide formed by the combination of a glucosyl and fructosyl moiety, sucrose is a major transport carbohydrate, transported from photosynthetic to non-photosynthetic cells (Geigenberger and Stitt, 2000; Salerno and Curatti, 2003; Rolland et al., 2006; Wind et al., 2010). Thus, sucrose acts as the primary energy source and as a plant growth and development signal (Eveland and Jackson, 2012; Lastdrager et al., 2014). However, only invertase (INV; IC 3.2.1.16) and sucrose synthase (SuSy; IC 2.4.1.13), have sucrose-catalyzing ability in plants. It is generally believed that INV hydrolyzes sucrose to glucose and fructose in the cell wall, vacuolar, and cytosolic fractions, whereas SuSy is localized in sink tissues and has reversible functions of both sucrose synthesis and degradation (Geigenberger and Stitt, 1991, 1993; Fernie et al., 2002; Koch, 2004; Rolland et al., 2006; Bieniawska et al., 2007; Angeles-Núñez and Tiessen, 2012; Eveland and Jackson, 2012). The structure of At.SuSy1 provides insight into its functions (Zheng et al., 2011), but it is unclear how SuSy actually affects sucrose metabolism.

Sucrose is a major photosynthetic product that is actively transported by the phloem and affects cell growth and division; thus, sucrose has a pivotal role in plant growth and development. Plant growth is a highly energy-demanding process that requires optimal sugar balance, particularly that of sucrose, between photosynthetic and non-photosynthetic cells. Numerous sugar signaling pathways have been identified which involve in the maintaining the balance between the sugar production and consumption, which helps avoid energy stress (Tiessen and Padilla-Chacon, 2013; Lastdrager et al., 2014). Starch regulates sugar status through biosynthesis and degradation during day and night, respectively (Chourey et al., 1998; Smith and Stitt, 2007; Angeles-Núñez and Tiessen, 2010; Graf and Smith, 2011; Farré and Weise, 2012). A relationship between sugar status and cell growth and development has been demonstrated in *Arabidopsis* via the SnRKs and TOR signaling pathways. SnRK1 is activated when plants have low sugar status (Chiou and Bush, 1998; Halford et al., 2003; Rolland et al., 2006; Coello et al., 2011), whereas TOR is activated in the presence of high levels of sucrose (Deprost et al., 2007; Robaglia et al., 2012; Lastdrager et al., 2014). Sucrose induces the expression of phytochrome-interacting factors (PIFs; Leivar and Quail, 2011), whereas degradation of PIFs is promoted by light-activated phytochromes (Castillon et al., 2007). This finding has helped bridge the gap to determine how plants alter growth through different day length (Nagel and Kay, 2012; Shin et al., 2013). Such sugar signaling pathways help to explain how plants sense and adapt to their energy source to regulate growth. However, there are still gaps in our understanding of how plants regulate and respond to sucrose level and the demand of sucrose flux to maintain the sucrose balance between photosynthetic and non-photosynthetic cells. In addition, changes in the morphology and development of plants occur after directly adding exogenous sucrose to plant culture media (Rolland et al., 2006; Wind et al., 2010; Liu et al., 2011; Eveland and Jackson, 2012); however, how plants regulate sucrose production and consumption for responses and the effects of increased endogenous sucrose on plant metabolism are poorly understood.

Plants possess pluripotent stem cells located in specialized regions called meristems that are capable of producing new cells to drive organogenesis. Stem cells are located in the center zone (CZ) of shoot apical meristems (SAMs) and receive energy (i.e., sucrose from source cells) and signals (e.g., phytohormones) to stimulate the production of new cells, thus making important contributions to plant growth and organogenesis. The populations of stem cells and their progenitors are tightly controlled during proliferation by a negative feedback loop between the WUSCHELL (WUS) transcription factor and the CLAVATA (CLV) pathway (Schoof et al., 2000; Grandjean et al., 2004; Traas and Bohn-Courseau, 2005; Williams and Fletcher, 2005; Francis and Halford, 2006). WUS promotes an increase in the number of stem cells, whereas the CLV pathway limits the number of stem cells by inhibiting WUS. The exogenous sucrose supply promotes *WUS* expression by stimulating cell division (Wu et al., 2005) and *CycD* expression (Riou-Khamlichi et al., 2000), which can increase cell division and, consequently, increase the number of stem cells, and thus plant development.

Several studies have suggested that heterologous overexpression of the *SuSy* gene in plants promotes the production of biomass (Coleman et al., 2006, 2009; Baroja-Fernández et al., 2009; Jiang et al., 2012; Xu et al., 2012; Li et al., 2013). These studies focused on changes in soluble sugars and biomass in ectopically expressed SuSy transgenic plants; however, the mechanism of how the changes in soluble sugars affect plant growth and development is poorly understood. Herein, we present the following results after transforming six *SuSy* genes (S1–S6) into *Nicotiana tabacum*: (1) sucrose-degrading sucrose synthase (SuSy) activity increased significantly in transgenic plants compared to that in wild-type (WT) plants; (2) total soluble sugars (TSS), particularly sucrose and fructose, increased markedly in the transgenic plants; and (3) increased chlorophyll content, a higher rate of photosynthetic efficiency, and the upregulated expression levels of the genes involved in the photosynthetic sucrose synthesis were observed in the transgenic plants compared to those in WT plants. These results suggest the existence of a novel sucrose signaling pathway. This novel signaling pathway involves unknown factors that stimulate photosynthesis in the photosynthetic cells, thereby securing the sucrose flux demanded from photosynthetic to non-photosynthetic cells. Consequently, the increase in sucrose upregulated the transcription of genes controlling growth, division, and elongation of stem cells in the SAM, resulting in pronounced changes in the development and phenotype of the transgenic plants.

## Materials and methods

### Plant materials and growth conditions for transformation

We used WT *Arabidopsis thaliana* Columbia ecotype (Col-0) and tobacco (*Nicotiana tabacum* SR1). WT Arabidopsis plants and tobacco seedlings were grown on MS medium (Murashige and Skoog, 1962) in culture room at 25±3°C under a 16 h/8 h light/dark photoperiod, and light intensity of 60 mmol m^−2^ s^−1^.

### Gene cloning, plasmid construction, plant transformation, and molecular analyses

We used the lithium chloride method and superscript reverse transcriptase (Invitrogen, Carlsbad, CA, USA) to obtain the mRNA and six full-length of *SuSy* genes from *A. thaliana* (Auffray and Rougeon, 1980) by using forward primer (FP) and reverse primer (RP; Supplementary Table S1). The cloned genes were inserted into pCambia 2300 with HA-NOS downstream, under regulation of the 35S promoter. *Agrobacterium tumefaciens* strain GV3013 was used for transformation of tobacco (*N. tabacum* cv. SR1) using the leaf disk method (Helmer et al., 1984).

Total genomic DNA was extracted from T_1_ generation transgenic plant leaves to confirm the presence of the heterologous *SuSy* in the transgenic plants, and the heterologous SuSy proteins were examined by Western blotting with a mouse HA antibody (LF-MA0048, Ab frontiers) as the primary antibody, and goat anti-mouse IgG (HRP, LF-SA5001, Ab frontiers) as the secondary antibody.

### Growth conditions, sampling, and phenotypic observations

At least 10 transgenic lines were confirmed in each SuSy transgenic plant (S1–S6). Three lines from each transgenic plant that had the highest sucrose-degrading SuSy activity were used for further analysis. Thirty individuals from each line were transferred to a greenhouse in 10-L pots in soil–perlite mixtures at 25±3°C under a 16 h/8 h light/dark photoperiod and light intensity of 100 mmol m^−2^ s^−1^. The first-day germination of each plant was recorded.

The tenth leaf from the top of ten individual of each chosen transgenic lines and those from WT plants were harvested after each 10 days, from 30 to 120 DAG, stored at −70°C and ground in liquid N_2_ to analyze SuSy enzymatic activities, soluble sugars, and chlorophyll contents.

The phenotypic characteristics of the transgenic and WT plants were also measured, and the carbohydrate content of each plant part was determined by gas chromatography (GC). A weight of 30 mg of each non- and popping-pretreated biomass samples were treated with 0.25 mL of 72% sulfuric acid (H_2_SO_4_) for 45 min at 30°C and diluted with 67.9 mL of distilled water to 4% H_2_SO_4_. Hydrolysis step was carried out at 121°C for 1 h in an autoclave machine. A solution containing a known amount of myo-inositol was used as an internal standard and was neutralized with ammonia solution by vortex mixing. Sodium borohydride solution (1 mL) and 0.1 mL of glacial acetic acid (18 M) were added to degrade the sodium tetrahydroborate. Next, 0.2 mL of methyl immidazol and 2.0 mL of anhydrous acetic acid were sequentially added. Finally, 5.0 mL of deionized water were added and extracted with 2.0 mL of dichloromethane. The samples were analyzed using GC (GC-2010; Shimadzu, Otsu, Japan) with a DB-225 capillary column (30 m × 0.25 mm i.d., 0.25 lm film thickness, J&W; Agilent, Folsom, CA, USA) operating with helium. The operating conditions were as follows: injector temperature of 220°C, flame ionization detector (FID) at 250°C, and an oven temperature of 100°C for 1.5 min with a constant increase of 5°C/min to 220°C.

A 2-cm long segment from the top of the shoot tip was harvested from the transgenic and WT plants (three individuals from each transgenic line and WT plants) to measure soluble sugars in the shot tip, at 60 DAG. All leaves at the shoot tip were eliminated, and only the stem including SAM was ground in N_2_ liquid. The powder was used to measure soluble sugars as mentioned below.

The experiment to demonstrate enhanced photosynthesis and photosynthetic sucrose synthesis was conducted as follow: Seeds from the three S1 transgenic lines which had the highest sucrose content and WT were sprayed in petri dishes with MS media without sucrose for seeding (kanamycin added for the transgenic). The dishes were placed in the dark (in a box) at 25±3°C until germination, and the germinated seeds were transferred immediately to new MS media without sucrose. The dishes were placed in the dark for the next 7 days. The transgenic and WT plants were sampled before the light treatment at eight DAG. After the light treatment started, whole seedlings samples (at least 0.5 g) were obtained every 6 h for 48 h and analyzed for chlorophyll, starch, and soluble sugar contents. The transgenic and WT seedlings were harvested after 48 h to extract RNA, synthesize cDNA, and examine *CHLG, SPS* and *SPP* genes expression levels by RT-PCR.

Different light/dark time treatment experiment was set up as described above, except that the seedlings were transferred to new MS without sucrose media after germination and exposed to different light/dark periods. The light/dark treatments were: L.3/D.21 (3 h in light/21 h in dark); L.6/D.18; L.9/D.15; L.12/D.12; and L.24/D.0 (full-time light continuously). The dishes were placed in the dark for 14 days after finishing their respective time for light treatment, and then transferred to the light again the next day. Samplings were obtained at three time points beginning on day 15: start of the light treatment, after the light treatment, and after the dark treatment, according to the assigned times. Fresh seedlings were harvested, ground in liquid N_2_, and the powder was used to analyze sucrose-degrading SuSy activity, starch and soluble sugar contents. Cross sections of stems and vertical sections of the shoot apical were prepared as described below for the light microscope analysis.

### Enzyme activity assay

The tenth leaf from the tops of three individuals from each chosen transgenic line and wild-type (WT) plants was harvested at 7:00 a.m. at 10 day intervals from 30 to 120 DAG, stored at –70°C and ground in liquid N_2_ to analyze SuSy, SPS, and SPP enzymatic activities, and soluble sugars.

SuSy enzymatic activity was determined through sucrose-degrading and sucrose-synthesizing SuSy activities, according to the previous methods (Geigenberger and Stitt, 1991, 1993; King et al., 1997; Hauch and Magel, 1998; Ruan, 2003; Koch, 2004; Bieniawska et al., 2007). Briefly, leaf powder was used to extract protein in a buffer containing of 50 mM Na_2_HPO_4_, pH 8.0, 1 mM EDTA, 5 mM DTT, 20 mM mercaptoethanol, and 10% glycerol. The triphenyltetrazolium chloride (TTC; ~95%; Cas. No. 298-96-4, Sigma Aldrich, USA) solution contained 0.25% (w/v) TTC, 1 M NaOH, and 0.08% Triton-X. The supernatant was collected and checked for total soluble protein by the Bradford method before measuring fructose produced in the reaction with TTC. Sucrose-degrading SuSy activity was determined by adding 10 μg protein from the supernatant to 200 μl of solution containing 10 mM sucrose and 10 mM UDP, pH 5.0, followed by a 1 h incubation at 37°C. Meanwhile, the same reaction without adding UDP was conducted to measure sucrose-degrading activity caused by other enzymes existing in the supernatant. Sucrose-synthesizing SuSy activity was determined by adding 10 μg proteins from the supernatant to 200 μl of solution containing 10 mM UDP-Glc and 10 mM fructose in the same condition. Control samples were conducted without adding the supernatant to detect fructose content. Fructose reacted with TTC and changes to a red color. Absorbance levels were obtained by measuring optical density (OD) at 495 nm by Multiskan EX spectrophotometer (ThermoScience, Rockford, IL, USA).

Sucrose phosphate synthase (SPS) enzymatic activity was measured by quantifying the sucrose fructosyl moiety using the anthrone test (Lunn and Furbank, 1997; Baxter et al., 2003), whereas sucrose phosphate phosphatase (SPP) was determined by following the release of orthophosphate from Suc6P (Ames, 1966; Lunn et al., 2000; Chen et al., 2005). SPS activity was determined by adding 10 μg protein of supernatant in a 200 μl buffer containing 50 mM HEPES–KOH pH 7.5, 20 mM KCl, and 4 mM MgCl_2_, 10 mM UDP-Glc and 10 mM Fru6P, incubated at 37°C for 1 h. The mixtures then were boiled for 5 min to stop the reaction before added 100 μl of 0.14% (w/v) anthrone reagent (in 14.6 M H_2_SO_4_), and absorbance was measured at OD 620 nm. SPP activity was determined by adding 10 μg protein of supernatant in a 200 μl the same buffer plus 1.25 mM Suc6P ~98% (Cas. No. 36064-19-4, Sigma Aldrich, USA) and incubated at 30°C for 1 h, and stopped by adding 30 μl of 2 M trichloroacetic acid. A 100 μl of 0.42% ammonium molybdate in 1 M H_2_SO_4_ was added, incubated for 10 min, and the solution was read at OD 820 nm.

### Soluble sugars and starch analyzes

The ground powder used to measure the enzymatic activities described above was used to analyze soluble sugar and starch contents. Sucrose, glucose and fructose were measured in 0.5 g of frozen powder that was re-suspended in 1 mL of ethanol 90%, left at 70°C for 90 min and centrifuged at 13,000 × g for 10 min. The supernatants were then filtered and subjected to high performance liquid chromatography with pulsed amperometric detection on a DX-500 Dionex system (Li et al., 2013). The pellet from the above sugar analysis was washed three times with ethanol 90% and then three times with distilled water before being assayed using an amyloglucosidase-based test kit (Sigma Aldrich, St. Louis, MO, USA) to determine starch content.

### Chlorophyll fluorescence and chlorophyll content

All six transgenic plant lines (S1–S6) were used to determine which has the highest sucrose content based on photosynthesis capability and chlorophyll content. Photosynthetic activity was measured at early morning in the tenth leaf from the top of the transgenic and WT plant was measured using the OS1-FL (OPTI—Sciences Co. Ltd., Hudson, NH, USA) as described previously (Hajirezaei et al., 2002). Chlorophyll was extracted from the frozen leaf powder using ethanol 90%, boiled for 5 min and measured absorbance at OD 620 nm to calculate chlorophyll concentration (Lichtenthaler, 1987).

### Light microscopy

Transgenic and WT 15 DAG seedlings were fixed in a freshly prepared mixed of 2% (w/v) glutaraldehyde and 4% (w/v) paraformaldehyde in 50 mM sodium cacodylate buffer (pH 7.4) to compare cell morphology. The samples were de-gassed and fixed under a vacuum for 4 h at room temperature. After washing in the same buffer, dehydration was done through a graded ethanol series. The specimens were then infiltrated and embedded in a LR-White resin (Wi et al., 2006), semi-thin sections (1–2 μm) were stained 1% toluidine blue and examined under a Carl Zeiss microscope (Axiolab; Carl Zeiss Inc., Jena, Germany).

Tobacco stems were fixed in FAA (formaldehyde, acetic acid, and ethanol) solution under vacuum to observe the general structure and starch distribution. After washing in distilled water, sections were cut on a rotary microtome using a disposable blade and stained with 1% toluidine blue and Lugol's solution (6 mM iodine, 42 mM KI, and 0.2 N HCl) to detect structure and starch granules, respectively (Hayashi et al., 2011).

## Results and discussion

### Soluble sugars, particularly sucrose, and sucrose-degrading SuSy activity increase in transgenic plants, probably due to enhanced photosynthesis and photosynthetic sucrose synthesis

Although it is generally accepted that SuSy possesses reversible functions, clarifying how this enzyme affects soluble sugars is complex. In particular, multi-gene families encoding SuSy isoforms exist in many plant species, but each of the six isoforms identified in *A. thaliana* has different expression patterns during development (Baud et al., 2004; Bieniawska et al., 2007). Six *At.SuSy* genes were heterologous overexpressed into *N. tabacum* to elucidate the function of SuSy isoforms in sucrose management and the mechanism by which soluble sugars, particularly sucrose, is affected. The six constructed vectors, each carrying one *SuSy* gene (*S1*–*S6*) driven by the 35S promoter, were introduced into tobacco plants by *Agrobacterium*-mediated transformation. Each successful transgene produced more than ten transgenic lines, and three were chosen for analysis. The constructed vectors and transgene expression levels were determined and shown in Supplementary Figures S1A,B.

**Figure 1:**
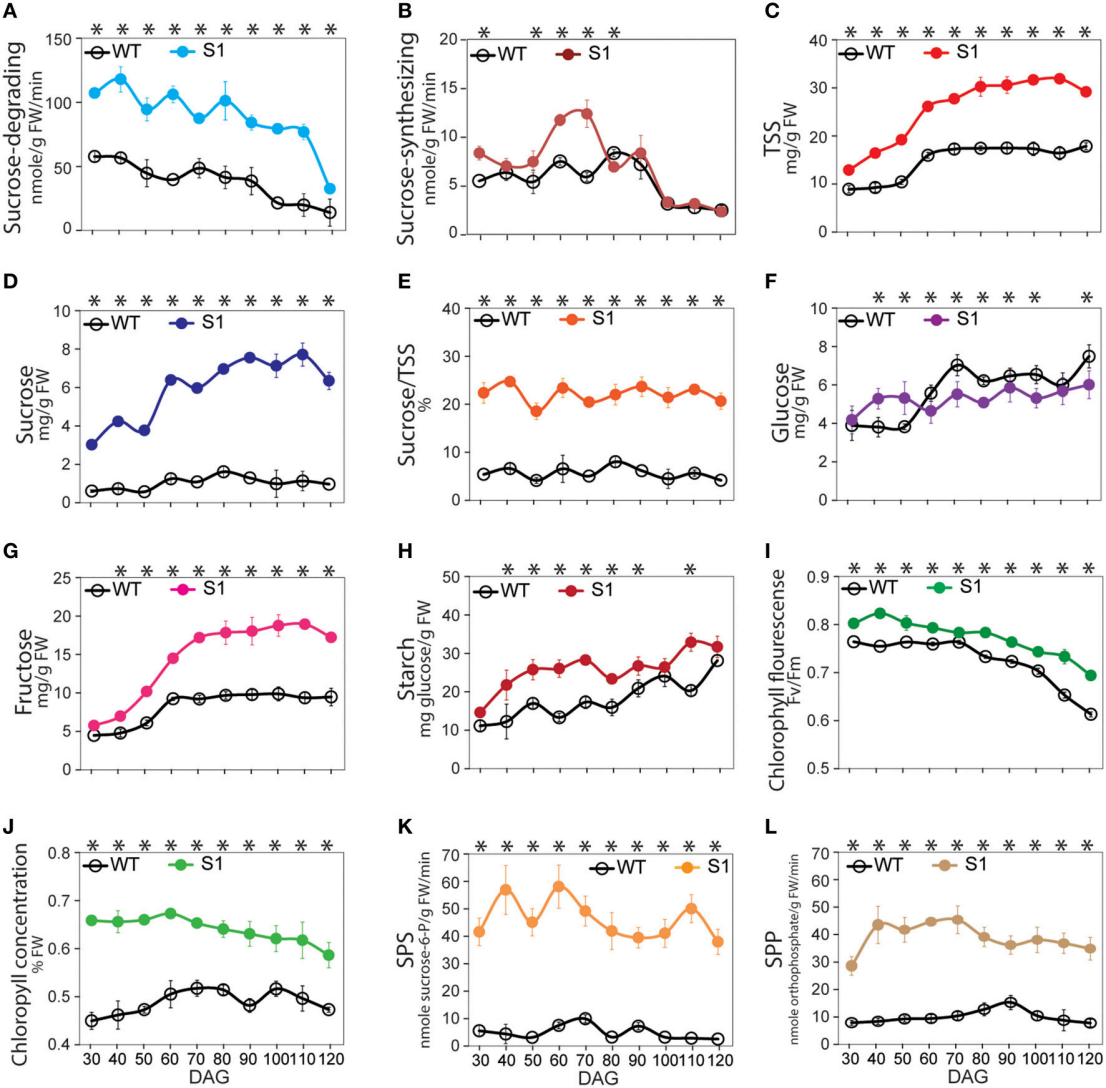
**Pattern of the sucrose synthase (SuSy) enzymatic activity, soluble sugar, starch, photosynthesis, chlorophyll concentration, sucrose-phosphate synthase (SPS) and sucrose-phosphatase (SPP) activity profiles of S1 transgenic and wild-type (WT) plants from 30 to 120 days after germination (DAG)**. **(A)** Sucrose-degrading SuSy activity. **(B)** Sucrose-synthesizing SuSy activity. **(C)** Total soluble sugar content (TSS), determined as the sum of sucrose **(D)**, glucose **(F)**, and fructose **(G)** contents. **(E)** Percentage of sucrose in TSS (%). **(H)** Starch content. **(I)** Photosynthetic capability, represented by chlorophyll fluorescence (Fv/Fm). **(J)** Chlorophyll concentration (% FW). **(K)** SPS activity. **(L)** SPP activity. Average values were calculated from triplicate (*n* = 3) from transgenic line which had highest sucrose-degrading SuSy activity. Asterisks (^*^) indicate significant differences from the control (WT), determined by a Student's *t*-test (^*^P < 0.05).

SuSy enzymatic activity was measured as sucrose-degrading and sucrose-synthesizing sucrose synthase (SuSy) activities. The sucrose-degrading and sucrose-synthesizing SuSy activity profiles revealed that successful heterologous overexpressed SuSy markedly upregulated sucrose-degrading SuSy activity in transgenic plants compared to in the WT plants (see Figure 1A for a comparison of S1 transgenic and WT plants and Supplementary Figure S1C for S1–S6 transgenic and WT plants), whereas low sucrose-synthesizing SuSy activity was detected, with no difference in the activity between WT and the transgenic plants (Figure 1B and Supplementary Figure S1E). Sucrose-degrading activities caused by other enzymes existing in the supernatant, including INVs, were low, and particularly, no different results between WT and the transgenic plants (Supplementary Figure S1D), suggested that others sucrose-degrading enzymes, particularly INVs did not involve in the upgraded sucrose-degrading activity. Sucrose-degrading SuSy activity was higher in shoots, stems, and roots of the transgenic plants than that in the WT (Supplementary Figure S1F), whereas no difference in sucrose-synthesizing SuSy activity was observed (Supplementary Figure S1G), indicating that the increase in sucrose-degrading SuSy activity occurred in all transgenic plants. Clearly sucrose-degrading SuSy activity, rather than sucrose-synthesizing SuSy activity, was exhibited in all heterologous overexpressed SuSy transgenic plants.

TSS, including soluble sucrose, glucose, and fructose, was distinctly elevated in the transgenic plants relative to that in the WT plants (Figure 1C and Supplementary Figure S1H), due to significant increase in sucrose and fructose (Figures 1D,G, respectively), whereas no difference in glucose was detected (Figure 1F). Consequently, sucrose made up the greatest proportion of TSS (Figure 1E), revealing that the TSS component has basically changed. The proportion of fructose was consistently higher in the transgenic than in the WT plants (Figure 1G), suggesting that increased sucrose in the transgenic plants may not be involved with sucrose-synthesizing SuSy activity. Sugar status can be influenced by starch biosynthesis and degradation (Smith and Stitt, 2007; Graf and Smith, 2011; Farré and Weise, 2012), but we found that starch increased significantly in the transgenic plants compared to the WT plants (Figure 1H), indicating that the increase in sucrose was not due to starch degradation. Notably, the same patterns were observed in sucrose and starch extracted from leaves, stems, and roots of the transgenic and WT plants (Supplementary Figures S1I,J, respectively), with higher sucrose-degrading SuSy activity occurring in these organs in the transgenic than in the WT plants. This demonstrates that the flux of sucrose delivery occurred at a higher rate in the transgenic than in the WT plants to distribute sucrose from photosynthetic to non-photosynthetic cells.

Autotroph means self-provided, thus, autotroph plants can synthesize almost all of their required carbohydrates from photosynthetically fixed carbon released from chloroplasts. The known sugar signaling pathways explain how plants control their growth to adapt to energy stress, but a mechanism must exist that allows plants to synthesize carbohydrates to compensate for any loss when they experience an unbalanced energy status. Analyses of the SuSy enzymatic activity, TSS, and starch patterns in the transgenic and WT plants at the same time after germination provided novel evidence that the increase in TSS, particularly sucrose, was not derived from either the sucrose-synthesizing SuSy activity or starch degradation, but was influenced by other factors that induced a series of reactions leading to the increase in sucrose. Before detecting the existence of the novel signaling pathway, we examined the directed responses that led to increased sucrose. We first proposed that the increased sucrose was driven by enhanced photosynthesis and photosynthetic sucrose synthesis. This enhancement of photosynthesis led to increased production of photosynthetically fixed carbon, such as TP in chloroplasts, which was delivered to the cytosol and utilized to synthesize sucrose, leading to overall enhancement of photosynthetic sucrose synthesis.

An abundance of chlorophyll in chloroplasts generally indicates proper photosynthesis (Beale, 1999; Bauer et al., 2001; Tanaka et al., 2001; Philippar et al., 2007; Moon et al., 2008; Shalygo et al., 2009; Berry et al., 2013). Of the six transgenic plants tested (S1–S6), the transgenic lines with the highest sucrose content were used to measure photosynthetic capability and chlorophyll content (see Materials and Methods). Consistent with our prediction, higher chlorophyll fluorescence (Fv/Fm) and chlorophyll concentration values were recorded in the transgenic lines (Figures 1I,J, respectively), indicating enhanced photosynthetic capability in the transgenic plants as compared to WT.

Enhanced photosynthetic efficiency leads to increased production of TP precursors for sugar synthesis (Bauer et al., 2001; Salerno and Curatti, 2003; Rolland et al., 2006). However, photosynthetic sucrose synthesis requires the participation of two other enzymes, sucrose phosphate synthase (SPS; E.C. 2.4.1.14) and sucrose phosphate phosphatase (SPP; EC 3.1.3.24; Rolland et al., 2006). Reverse-genetic approaches have demonstrated that reduced expression of these enzymes inhibits photosynthesis, indicating that sugar metabolism can also regulate the photosynthesis through downstream signaling (Baxter et al., 2003; Prasad et al., 2004; Chen et al., 2005) because decreased expression of these genes leads to a surplus of TP, followed by inhibited chloroplast function to cope with the sugar demand. We hypothesized that the increase in TP driven by enhanced photosynthesis upregulates *SPS* and *SPP*. Some studies have demonstrated an association between increased CO_2_ increased photosynthesis, and *SPS* expression (Farrar and Williams, 1991; Hussain et al., 1999; Vu et al., 2001; Prasad et al., 2004). Moreover, although *SPS* and *SPP* expression are regulated by day/night length (Huber and Huber, 1996; Chen et al., 2005; Sun et al., 2011), a clear correlation between enhanced photosynthesis and the expression levels of these genes is still missing. Herein, SPS and SPP activities were significantly higher in the transgenic than in the WT plants (Figures 1K,L, respectively, and Supplementary Figures S1K,L), in accordance with our hypothesis. These results indicate that *SPS* and *SPP* were upregulated in the transgenic plants.

To summarize, combined with the increased sucrose levels shown above (Figures 1D,E), our data suggest that enhanced photosynthesis and photosynthetic sucrose synthesis occur as a response in the condition of the increased sucrose-degrading SuSy activity in the transgenic plants.

### Increased endogenous sucrose content may upregulate of *WUS* and *CycD3*, which induce the SAM and trigger pronounced phenotypic and morphological changes in transgenic plants

**Figure 2:**
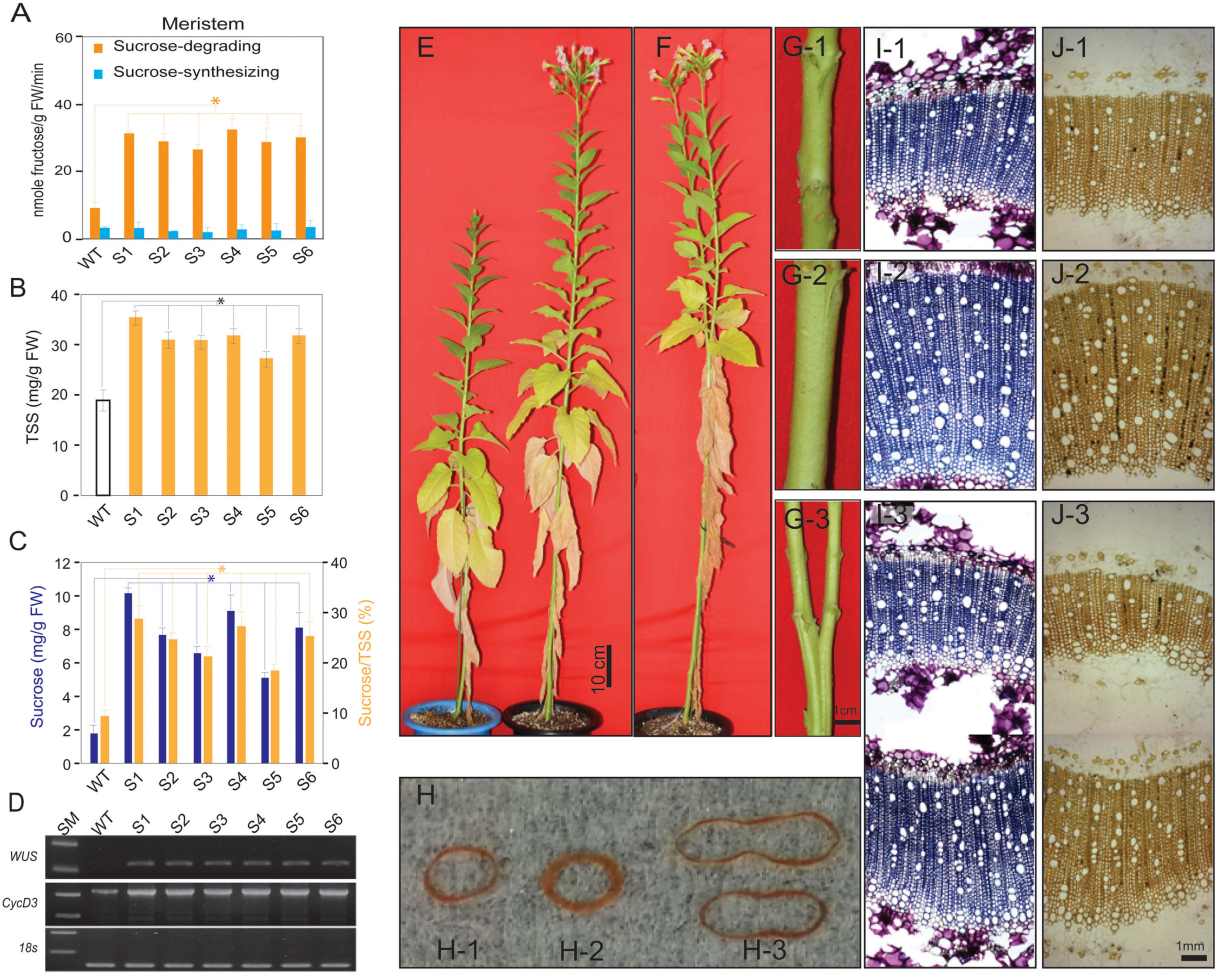
**Analysis of sucrose synthase activities, sugars, *WUSCHELL* (*WUS*) and *CycD3* expression levels in shoots of S1–S6 transgenic and wild-type (WT) plants, and the pronounced phenotypic changes in the transgenic plants relative to those in WT plants**. **(A)** Sucrose-degrading and sucrose-synthesizing SuSy activities in the shoot apical meristem of WT and transgenic tobacco plants. **(B)** Total soluble sugar (TSS) and **(C)** sucrose contents and concentrations, respectively. **(D)** *WUS* and *CycD3* expression levels. **(E)** The S1 transgenic plants developed faster than WT plants, as represented by higher height and quicker flowering. **(F)** Bifurcated stems in S1 transgenic plants. **(G)** Stem diameter of WT **(G-1)**, S1 transgenic **(G-2)**, and bifurcated stem of S1 transgenic plant **(G-3)**. **(H)** Xylem layer of WT **(H-1)**, S1 transgenic **(H-2)**, and bifurcated stem of S1 transgenic plant **(H-3)**. **(I)** Microtome stems structure showing xylem layer of WT **(I-1)**, S1 transgenic **(I-2)**, and S1 transgenic bifurcated stem **(I-3)**. **(J)** Microtome stem structure showing starch granules distributed in xylem layer of WT **(J-1)**, S1 transgenic **(J-2)**, and S1 transgenic bifurcated stem **(J-3)**. Average values were calculated from triplicate (*n* = 3) from transgenic line which had highest sucrose-degrading SuSy activity. Asterisks (^*^) indicate significant differences from the control (WT), determined by a Student's *t*-test (^*^P < 0.05).

All aboveground plant tissues and organs are derived from the SAM, which is located in the shoot tips and harbors sets of pluripotent stem cells embedded in the CZ. The number of stem cells and their progeny population, which is tightly regulated by energy signals, determine the size of the CZ (Bodson and Outlaw, 1985; Medford et al., 1991; Pien et al., 2001). Here, we wanted to link the increase in sucrose to the remarkable SAM development of transgenic plants than in the WT plants. Samples were harvested from the shoot tips of the transgenic and WT plants 60 days after germination (DAG) to measure soluble sugar. Significant higher in sucrose-degrading SuSy activity, but low and no difference in sucrose-synthesizing SuSy activity were obtained in the shoot tips of the transgenic and WT plants (Figure 2A). The SuSy transgenic plants contained more TSS (35.5 mg/g FW in S1 compared to 19.0 mg/g FW in WT plants, Figure 2B), with significantly higher sucrose content (10.2 mg/g FW in S1 compared to 1.7 mg/g FW in WT plants), which led to a higher proportion of sucrose in the TSS in the transgenic plants than in the WT plants (about 28.9% in S1 compared to 9.4% in WT plants, Figure 2C). Sucrose and fructose contents were also higher in the transgenic than in the WT plants (Supplementary Figure S2A). These data are similar to the results above, and show basic changes in the TSS component in the SAM, with higher sucrose in the transgenic than WT plants, derived from photosynthetic cells via sucrose flux demanded.

Stem cells in the SAM are controlled by WUS and exogenous sucrose promotes *WUS* expression by stimulating cell division (Wu et al., 2005) and inducing the expression of *CycD* genes, leading to increased cell division (Gaudin et al., 2000; Riou-Khamlichi et al., 2000). Our results show an abundance of endogenous sucrose in the SAM of the transgenic plants, which may affect *WUS* and *CycD3.1* expression levels. Reverse transcription–polymerase chain reaction (RT–PCR) analysis revealed dramatic upregulation of relative *WUS* and *CycD3.1* expression in the S1 transgenic plants compared to that in the WT plants (Figure 2D).

Our data suggest that the abundance of endogenous sucrose driven by enhanced photosynthesis and photosynthetic sucrose synthesis was sufficient to induce the expression of genes controlling stem cell development, which lead to pronounced changes in the phenotypes of the transgenic plants; i.e., longer stem height (Figure 2E and Supplementary Figure S2B), thicker stem diameter (Figure 2G and Supplementary Figure S2C), shorter time to generate the initial bud and to flowering (Table 1), and enhanced reproduction and biomass production (Table 2). These results are similar to those of previous studies, which demonstrated the effects of exogenous sucrose supply on plant development and physiology. For example, a study (Bodson and Outlaw, 1985) reported that the accumulation of sucrose in the meristem is an early physiological event in floral transition, which was later reviewed (Bernier et al., 1993), who hypothesized a regulatory loop between sucrose and cytokinins to control the transition to flowering at the SAM. Later, a study stated that sugar signals may also regulate meristematic proliferation at the G2 to M transition (Skylar et al., 2011). In our study, the secondary xylem was thicker in the transgenic than in the WT plants (Figure 2G). Particularly, bifurcated stems (Figure 2F) formed in all transgenic lines (more than 30% of all six transgenic plants had bifurcated stems, Supplementary Figure S2D), and xylem morphology was also affected (Figures 2H,I). The first demonstration about the formation of bifurcated stems and the involvement of an oligosaccharide signal molecule to the formation was introduced without a clear mechanism (Schmidt et al., 1993). We observed these features in all six transgenic lines tested, suggesting that the stem cells and SAM structural development patterns were altered by the increased endogenous sucrose content. More predominant starch granules were observed in the stem section of transgenic plants, clearly indicating that starch content was also higher in the transgenic than in WT plants (Figure 2J).

**Table 1:** **Time required for germination to initial bud generation (BG), initial flowering (F), and from BG to F in the wild-type (WT) and transgenic plants**.

	**Time required (DAG)**		
**Line**	**Initial buds generation (BG)**	**Initial flowering (F)**	**From BG to F**
WT	124.1 ± 4.7	137.8 ± 3.9	13.7 ± 2.1
S1	97.5 ± 4.1^*^	108.6 ± 3.5^*^	11.1 ± 1.0^*^
S2	101.4 ± 3.4^*^	113.4 ± 3.1^*^	12.0 ± 2.0^*^
S3	102.1 ± 3.1^*^	114.6 ± 4.4^*^	12.3 ± 1.5^*^
S4	102.6 ± 3.7^*^	115.8 ± 4.6^*^	12.6 ± 1.2^*^
S5	99.6 ± 2.9^*^	112.4 ± 1.7^*^	12.9 ± 2.0^*^
S6	102.8 ± 3.1^*^	116.8 ± 3.1^*^	12.6 ± 1.3^*^

Mean values were calculated from data obtained from individuals (n = 10) in each chosen transgenic line and WT. Asterisks (

* ) indicate significant differences determined by a Student's t-test (^*^P < 0.05).

**Table 2:** **Comparison of reproduction and biomass weight characteristics between the wild-type (WT) and transgenic plants**.

**Line**	**SuSy-degrading activity at flowering (nmol fructose/g FW/min)**	**Phenotype**						
		**Number of buds/flowers (unit)**	**Total harvested seed weight (g/plant)**	**100-seeds weight (mg/100 seeds)**	**After freeze drying—Weight (g/plant)**			
					**Leaves**	**Stem**	**Roots**	**Total dried biomass**
T	23.2 ± 1.2	38.8 ± 4.3	1.1 ± 0.0	11.0 ± 0.1	7.2 ± 0.9	6.9 ± 1.1	2.5 ± 0.5	16.7 ± 1.5
S1	60.2 ± 4.6^*^	50.7 ± 6.1^*^	1.8 ± 0.1^*^	12.0 ± 0.1^*^	9.8 ± 0.0^*^	12.9 ± 0.1^*^	5.2 ± 0.3^*^	28.0 ± 0.2^*^
S2	54.5 ± 3.9^*^	47.1 ± 4.3^*^	1.3 ± 0.0^*^	11.8 ± 0.2^*^	9.6 ± 0.6^*^	11.3 ± 0.8^*^	3.9 ± 0.0^*^	24.8 ± 1.5^*^
S3	67.4 ± 2.4^*^	46.1 ± 2.9^*^	1.3 ± 0.1^*^	11.7 ± 0.4^*^	9.6 ± 0.4^*^	10.9 ± 0.6^*^	5.0 ± 0.2^*^	25.5 ± 0.0^*^
S4	53.8 ± 3.5^*^	49.3 ± 2.5^*^	1.6 ± 0.1^*^	12.0 ± 0.1^*^	9.9 ± 0.0^*^	11.5 ± 1.4^*^	6.1 ± 0.2^*^	27.7 ± 1.2^*^
S5	69.2 ± 1.0^*^	48.6 ± 4.0^*^	1.5 ± 0.2^*^	11.7 ± 0.3^*^	9.6 ± 0.3^*^	10.9 ± 0.3^*^	5.4 ± 0.0^*^	26.0 ± 0.6^*^
S6	50.2 ± 1.3^*^	48.8 ± 3.8^*^	1.5 ± 0.2^*^	11.8 ± 0.3^*^	9.7 ± 0.6^*^	11.3 ± 0.3^*^	4.2 ± 0.0^*^	25.3 ± 0.9^*^

Mean values were calculated from the data obtained from individuals (n = 10) of each chosen transgenic line and WT. Asterisks (

* ) indicate significant differences determined by a Student's t-test (^*^P < 0.05).

Plant biomass was harvested and analyzed by gas chromatography (GC) to examine carbohydrates that accumulated in the plant cell walls after complete maturation (Coleman et al., 2009). The argument was that different SuSy isoforms can have different effects on carbohydrate accumulation in the cell wall. The results showed higher total carbohydrate contents in all parts of the plant (leaves, stems, and roots) in the transgenic than in WT plants (Table 3). The highest glucose content was observed in leaves, compared to that in stems and roots. Glucose was significantly higher in the transgenic than in WT plants, whereas xylose was lower in leaves but higher in the stems and roots. These data confirm no differences in the effects of the SuSy isoforms on carbohydrate accumulation in plant cell walls, as all of the isoforms revealed altered carbohydrate content in the transgenic plants, consistent with previous studies (Coleman et al., 2009; Jiang et al., 2012; Xu et al., 2012; Li et al., 2013).

**Table 3:** **Comparison of carbohydrate content in leaves, stem, and roots between the wild-type (WT) and transgenic plants**.

	**(%)**	**WT**	**S1**	**S2**	**S3**	**S4**	**S5**	**S6**
Leaves	Rhamnose	2.4±0.6	2.7±0.7^*^	2.7±0.5^*^	2.6±0.6^*^	2.3±0.3	2.4±0.4	2.5±0.5
	Arabinose	1.2±0.1	1.3±0.4	1.4±0.1	1.3±0.0	1.1±0.1	1.2±0.1	1.0±0.1
	Xylose	1.5±0.3	1.7±0.2^*^	1.6±0.1	1.4±0.0	1.2±0.1^*^	1.3±0.1^*^	1.1±0.0^*^
	Mannose	0.9±0.4	1.3±0.7	0.9±0.4	0.9±0.1	0.8±0.2	0.9±0.2	0.8±0.0
	Galactose	2.8±0.2	2.8±1.1	3.1±0.5^*^	2.8±0.4	2.6±0.3^*^	2.7±0.5	2.3±0.1^*^
	Glucose	46.5±1.2	62.6±4.4^*^	55.5±1.0^*^	50.5±0.8^*^	49.8±2.2	47.0±1.1	45.5±2.4
	Total	55.4±5.6	72.5±8.4^*^	65.3±4.4^*^	59.4±4.4^*^	57.8±2.4	55.5±6.1	53.3±2.7
Stems	Rhamnose	1.8±0.4	1.3±0.1^*^	1.3±0.4^*^	1.2±0.2^*^	1.1±0.2^*^	1.2±0.1^*^	1.3±0.2^*^
	Arabinose	0.7±0.2	0.6±0.0	0.7±0.0	0.7±0.0	0.6±0.0	0.6±0.1	0.5±0.1
	Xylose	5.6±0.9	8.2±1.5^*^	8.4±0.6^*^	9.7±1.1^*^	9.8±0.1^*^	9.0±0.9^*^	8.8±0.9^*^
	Mannose	1.0±0.2	1.4±0.3^*^	1.4±0.3^*^	1.6±0.0^*^	1.6±0.1^*^	1.7±0.1^*^	1.5±0.1^*^
	Galactose	1.5±0.1	1.3±0.2	1.3±0.2	1.3±0.1	1.1±0.1^*^	1.1±0.1^*^	1.0±0.2^*^
	Glucose	43.5±1.2	50.9±6.1^*^	49.7±5.7^*^	52.8±2.9^*^	52.7±2.9^*^	46.9±3.6^*^	46.2±2.5
	Total	54.2±3.4	63.6±4.5^*^	62.7±5.4^*^	67.3±1.1^*^	67.0±3.3^*^	60.4±3.6^*^	59.4±1.5^*^
Roots	Rhamnose	1.2±0.6	1.2±0.1	1.3±0.3	1.4±0.3^*^	1.3±0.1	1.0±0.2^*^	1.2±0.5
	Arabinose	0.7±0.2	0.9±0.1^*^	0.9±0.0^*^	1.0±0.1^*^	0.9±0.1^*^	0.9±0.2^*^	1.0±0.1^*^
	Xylose	6.4±3.5	9.1±1.1^*^	9.8±1.1^*^	8.8±0.3^*^	8.1±0.7^*^	6.9±0.4	8.9±1.9^*^
	Mannose	1.1±0.5	1.1±0.1	0.9±0.0	0.9±0.0	0.8±0.0	0.7±0.0^*^	0.9±0.2
	Galactose	1.3±0.1	1.0±0.3^*^	1.0±0.1^*^	1.2±0.0	1.0±0.2^*^	1.1±0.1^*^	1.3±0.3
	Glucose	42.0±3.8	47.7±2.6^*^	46.1±1.3^*^	50.0±1.5^*^	48.9±4.1^*^	45.1±1.8^*^	51.4±7.8^*^
	Total	52.7±3.6	61.1±6.4^*^	60.0±3.4^*^	63.2±2.4^*^	61.0±4.5^*^	55.8±3.5^*^	64.7±4.6^*^

Mean values were calculated from the data obtained from individuals (n = 3) of each chosen transgenic line and WT. Asterisks (

* ) indicate significant differences determined by a Student's t-test (^*^P < 0.05).

### Evidence for a novel sucrose signaling pathway leading to enhanced photosynthesis and photosynthetic sucrose synthesis

Sucrose is a photosynthetic product, produced in photosynthetic cells and transported to non-photosynthetic cells, and the synthesis, transport from source to sink cells, and use of sucrose are tightly regulated (Koch, 2004; Rolland et al., 2006; Wind et al., 2010). A proper balance between hexose (glucose and fructose) and sucrose is required for normal plant development. As reported previously, expression of *SnRKs* genes is upregulated in culture media without sucrose, whereas *TOR* genes are upregulated in media with an abundance of sucrose (Chiou and Bush, 1998; Halford et al., 2003; Rolland et al., 2006; Coello et al., 2011; Robaglia et al., 2012; Lastdrager et al., 2014). In contrast, our hypothesis about the existence of an unknown sucrose signaling pathway is strongly supported by the higher levels of TSS and each of its sugars, as well as enhanced photosynthesis and photosynthetic sucrose synthesis leading to increased sucrose content. We conducted an experiment to detect the relationship between the transition from heterotrophic to autotrophic growth and the variations in chlorophyll content and soluble sugars to determine the existence of a sucrose signaling pathway. Accordingly, when the carbohydrate reserved in the seed is depleted, seedlings must synthesize their own carbohydrates (Baker et al., 2006; Rolland et al., 2006; Stitt and Zeeman, 2012). We also conducted an experiment to analyze the changes when plants are transferred from dark to light conditions. Harvested samples were analyzed for chlorophyll, starch, and soluble sugar contents, and *CHLG, SPS* and *SPP* gene expression levels were determined by RT-PCR after 48 h.

**Figure 3:**
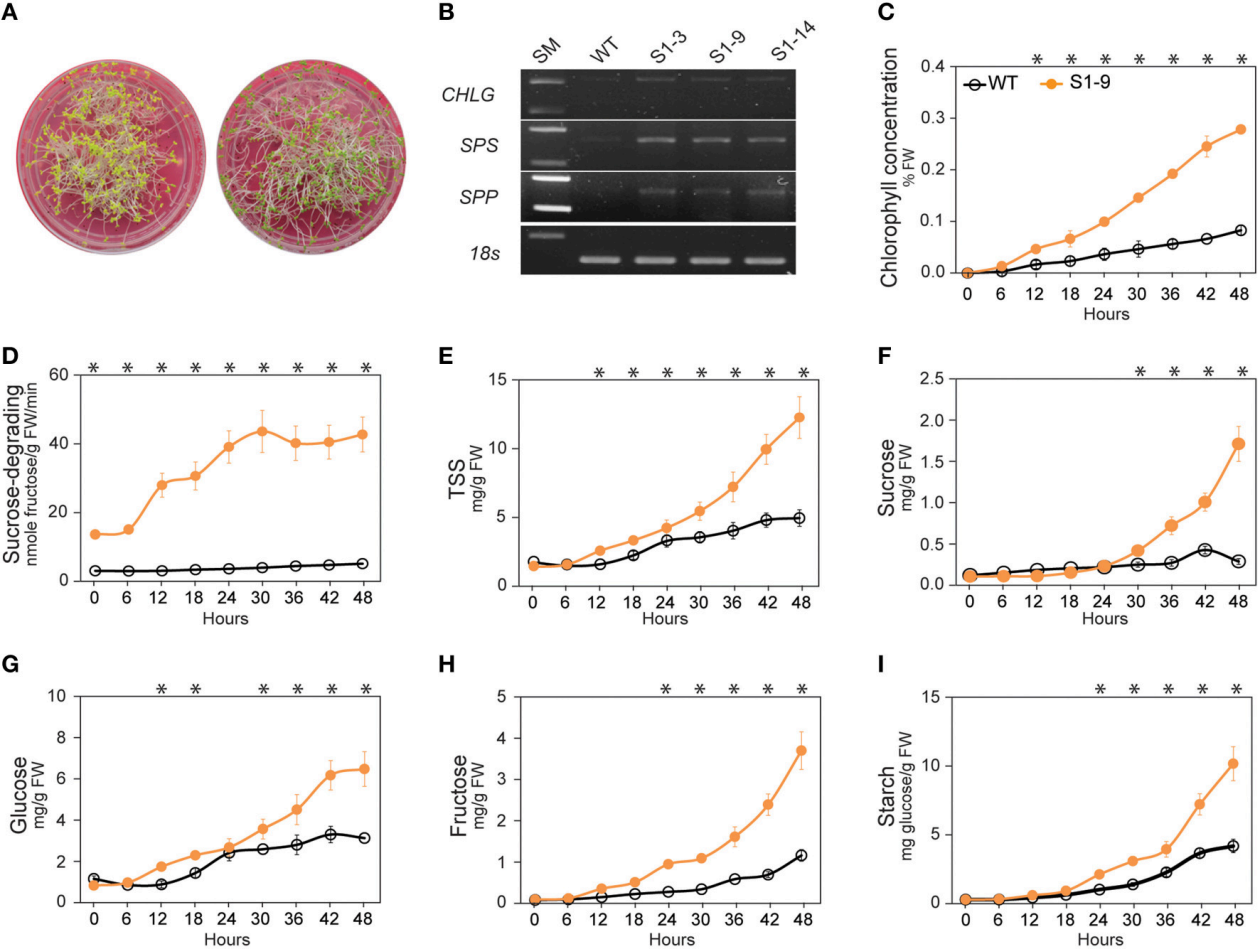
**Results of experiment demonstrating enhanced photosynthesis and photosynthetic sucrose synthesis in sucrose synthase (SuSy) transgenic plants**. **(A)** Leaves of S1 transgenic seedlings turned nearly green color, whereas WT seedlings remained yellowish after the 48 h light treatment. **(B)** *Chlorophyll synthase* (*CHLG), sucrose-phosphate synthase* (*SPS*) and *sucrose-phosphatase* (*SPP*) expression levels after the 48 h light treatment. **(C)** Pattern of chlorophyll concentration (% FW), 6 h intervals from 0 to 48 h after starting the light treatment. **(D)** Sucrose-degrading SuSy activity. **(E)** Total soluble sugar (TSS) content, represented by the sum of content of sucrose **(F)**, glucose **(G)**, and fructose **(H)** contents. **(I)** Starch content. Average values were calculated from triplicate (*n* = 3). Asterisks (^*^) indicate significant differences from the control (WT), determined by a Student's *t*-test (^*^P < 0.05).

As shown in Figure 3A, the leaves of the S1 transgenic seedlings turned nearly green, whereas leaves of the WT plants remained yellowish after 48 h of light treatment. *CHLG, SPS*, and *SPP* expression levels were also higher in the S1 transgenic seedlings than in the WT plants (Figure 3B), reinforcing our hypothesis. Chlorophyll concentration (% FW) revealed that chlorophyll was synthesized faster after light treatment (Figure 3C), suggesting that the setup or recovery of photosynthetic capacity within 48 h was stronger in the S1 transgenic than in the WT seedlings. In addition, sucrose-degrading SuSy activity was also higher in the S1 transgenic than in the WT seedlings (Figure 3D). Almost no difference in TSS content were observed between transgenic and WT seedlings for light treatments of 0–6 h, whereas TSS in the S1 transgenic seedlings was clearly higher than that in the WT seedlings beginning at 12 h (Figure 3E). In more detailed, sucrose increased slowly in the WT seedlings during the first 24 h of the light treatment, but we found almost no change (or slight reduction) in the transgenic seedlings, which can be explained by the sucrose-degrading function of heterologous S1; however, sucrose increased strongly beginning at 24 h of light treatment in the transgenic seedlings relative to in the WT seedlings (Figure 3F). It appears that glucose increased by 36 h of the light treatment (Figure 3G). Fructose and starch also increased (Figures 3H,I, respectively), strengthen the argument that the increased sucrose was not derived from either degradation of starch or sucrose synthesis with fructose as precursor. These data suggest that the sucrose signaling pathway induces the prompt setup or recovery of photosynthesis and photosynthetic sucrose synthesis, leading to the impressive increase in TSS, particularly sucrose, observed in the transgenic plants. Moreover, the sucrose signaling pathway was activated soon after transferring the seedlings from heterotrophic to autotrophic growth conditions (from dark to light treatment), which caused a dramatic shift in the photosynthetic setup. Once the setup was completed, chlorophyll was synthesized, and photosynthetic sucrose synthesis started in the photosynthetic cells.

**Figure 4:**
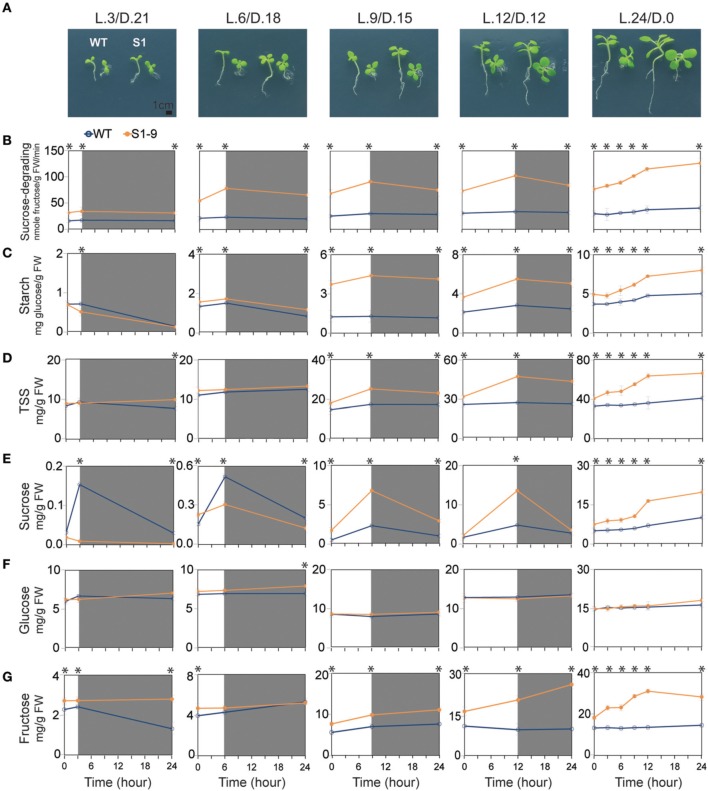
**Different phenotypes and sucrose-degrading sucrose synthase (SuSy) activity pattern, starch and sugar contents in the transgenic and wild-type (WT) seedlings exposed to different light/dark time treatment**. **(A)** Transgenic seedlings developed faster than wild-type (WT) seedlings under all light/dark treatments, as represented by larger transgenic than WT seedling (bar = 1 cm). Detailed are shown detailed in Supplementary Table S2. **(B)** Sucrose-degrading SuSy activity. **(C)** Starch content. **(D)** Total soluble sugar (TSS) content, represented by the sum of sucrose **(E)**, glucose **(F)**, and fructose **(G)** contents. Average values were calculated from triplicate (*n* = 3). Asterisks (^*^) indicate significant differences from the control (WT), determined by a Student's *t*-test (^*^P < 0.05).

Sugar metabolism in plants is regulated primarily by light/dark duration. Synthesis of soluble sugars, particularly sucrose, and starch occur by photosynthesis in the presence of light, and decomposition occurs during respiration in the dark. Starch synthesis and degradation are key mechanisms used to maintain the balance of sugars (Smith and Stitt, 2007; Kötting et al., 2010; Graf and Smith, 2011; Farré and Weise, 2012; Stitt and Zeeman, 2012). Thus, we conducted an experiment with variable light/dark time to determine the influence of different day lengths on sugar metabolism, how variations in sugars affects plant growth and development, and how increased sucrose under light conditions may not be driven by starch degradation in the SuSy transgenic plants. Seedlings were transferred to new MS without sucrose media and exposed to different light/dark periods. The light (L)/dark (D) treatments were: L.3/D.21 (3 h light/21 h dark); L.6/D.18; L.9/D.15; and L.12/D.12. The dishes were put in the dark after completing the respective times under light treatment for 14 days and then transferred to light again the next day. Plants exposed to L.24/D.0 were given light all the time. Samples were collected at three time points starting day 15: start of the light treatment, after the light treatment, and after the dark treatment, according to the assigned times. The samples were analyzed for sucrose-degrading SuSy activity and starch and soluble sugar contents. The fresh samples were photographed to compare phenotypes and to analyze stem cross and SAM vertical sections. Evaluation of seedlings development revealed that the longer light treatment resulted in faster development and faster growth rates in the S1 transgenic plants compared to those in the WT seedlings (Figure 4A). The phenotypic characteristics were also higher in the S1 transgenic plants than those in the WT for the same setting (Table S2). Stem xylem layer morphology varied based on the cross-sections (Supplementary Figure S3A), indicating that a higher growth rate was achieved with longer photosynthesis times in the transgenic than in the WT plants. Thicker xylem layers in the transgenic plants exposed to L.9/D.15, L.12/D.12, and L.24/D.0, suggested improved secondary xylem formation, derived from the primary xylem. Vertical sections revealed differences in the size of the shoot apical structure, as evidenced by longer vertical and horizontal lines. Calculations using Photoshop CS6 software showed that the shoot apical area was larger in the S1 transgenic than in the WT plants (Supplementary Figures S3B,C), indicating enhanced development of the SAM in the transgenic plants, which helps explain the faster vegetative growth of the transgenic plants.

Sucrose-degrading SuSy activity showed an increasing trend during light treatment, but a decreasing trend during the dark treatment, with higher sucrose-degrading SuSy activity achieved during the longer light treatment times. These features were clearly observed in the transgenic plants exposed to L.24/D.0, indicating that heterologous *SuSy* isoforms were induced during photosynthesis (Figure 4B). The same patterns were also observed for starch content (Figure 4C). Significantly higher rates of synthesized and total starch content (about 3.4 times higher; 4.4 mg glucose/g FW in S1 transgenic compared to 1.3 mg glucose/g FW in WT seedlings after 9 h, and about twice higher; 5.5 mg glucose/g FW in S1 transgenic compared to 2.8 mg glucose/g FW in WT seedlings after 12 h of light treatment, respectively) were detected after the light treatment in transgenic seedlings exposed to L.9/D.15 and L.12/D.12 than in the WT seedlings. Seedlings exposed to L.24/D.0 showed a 63% increase in starch (from 4.9 to 8.0 mg glucose/g FW) in the S1 transgenic, compared to 37% (from 3.7 to 5.0 mg glucose/g FW) in the WT seedlings after 24 h of light treatment. We found no difference in starch degradation rate after the dark treatment, but starch content was higher in the transgenic than in WT seedlings. These data suggest that, degradation of starch is not involved in the increase of sucrose during the light treatment, as presented below.

The variations in soluble sugars are shown in Figures 4D–G, and sucrose revealed the most interesting results. Small quantities of sucrose were detected in the S1 transgenic and WT seedlings after 3 and 6 h of light during the day, but the respective trends were differed. The WT seedlings tended to increase sucrose, whereas the transgenic seedlings showed a decreasing trend, clearly indicating the function of upregulated sucrose-degrading SuSy activity in the transgenic seedlings. However, we found significantly more sucrose in transgenic than in the WT seedlings after 9 and 12 h of light during the day. About three times more (6.8 mg/g FW in S1 transgenic compared to 2.3 mg/g FW in WT seedlings) was detected after light treatment for 9 h, and 2.9 times more (13.5 mg/g FW in S1 transgenic compared to 4.7 mg/g FW in WT seedlings) were detected after 12 h of light (Figure 4E), which contributed to the dramatic increase in TSS in the transgenic relative to that in the WT seedlings (Figure 4D). As mentioned, starch was also significantly higher in the samples (Figure 4C), confirming that the increased sucrose was not the result of starch degradation. Sucrose tended to decrease after the dark treatment in both the transgenic and WT seedlings, but the rate of decrease was significantly higher in the transgenic than in WT seedlings, suggesting that the ectopically expressed SuSy properly displayed a sucrose-degrading SuSy function after photosynthetic sucrose synthesis was interrupted. The results also showed that, about two times more sucrose was produced in the exposed to L.24/D.0 transgenic than in the WT seedlings (19.7 mg/g FW in the S1 transgenic compared to 9.9 mg/g FW in the WT; Figure 4E). These data suggest that photosynthetic sucrose synthesis was strongly induced in the transgenic seedlings receiving light for 9 h/day, which was closely related to upregulated SPS and SPP activities in the source cells (Figures 1J,K). This resulted in the production of large amounts of sucrose. In addition, insufficient amounts of sucrose in the transgenic seedlings seemed to be due to the effects of ectopically expressed SuSy, shown by the reduced quantity of sucrose produced in a plants exposed to short day length. Similarly, a significantly higher quantity of fructose was detected in the transgenic than in WT seedlings: about 1.4 times higher (9.9 mg/g FW in S1 transgenic compared to 7.0 mg/g FW in WT seedlings), and about 2.2 times higher (20.4 mg/g FW in S1 transgenic compared to 9.4 mg/g FW in WT seedlings), after 9 and 12 h of light/day, respectively (Figure 4G), but no difference in glucose was observed (Figure 4F). The data from different light/dark treatment experiment show that variations in sucrose indicate the existence of a sucrose signaling pathway and show how seedlings are influenced by different durations of photosynthesis.

In summary, we first demonstrated that the increase in sucrose during upregulated sucrose-degrading SuSy activity was directly due to enhanced photosynthesis and photosynthetic sucrose synthesis. However, these improvements were triggered and driven by an unknown sucrose signaling pathway that exists in the condition of the more abundant of SuSy protein is presented in whole organs of the transgenic tobacco plants. We propose that the sucrose signaling pathway triggers the following photosynthetic response cells in order: (1) acceleration of photosynthetic efficiency by increasing chlorophyll synthesis enhances the production and releases of TP from chloroplasts into the cytosol; and (2) the abundance of TP leads to upregulated photosynthetic sucrose synthesis by enhancing *SPS* and *SPP* transcriptional levels, leading to increased sucrose.

**Figure 5:**
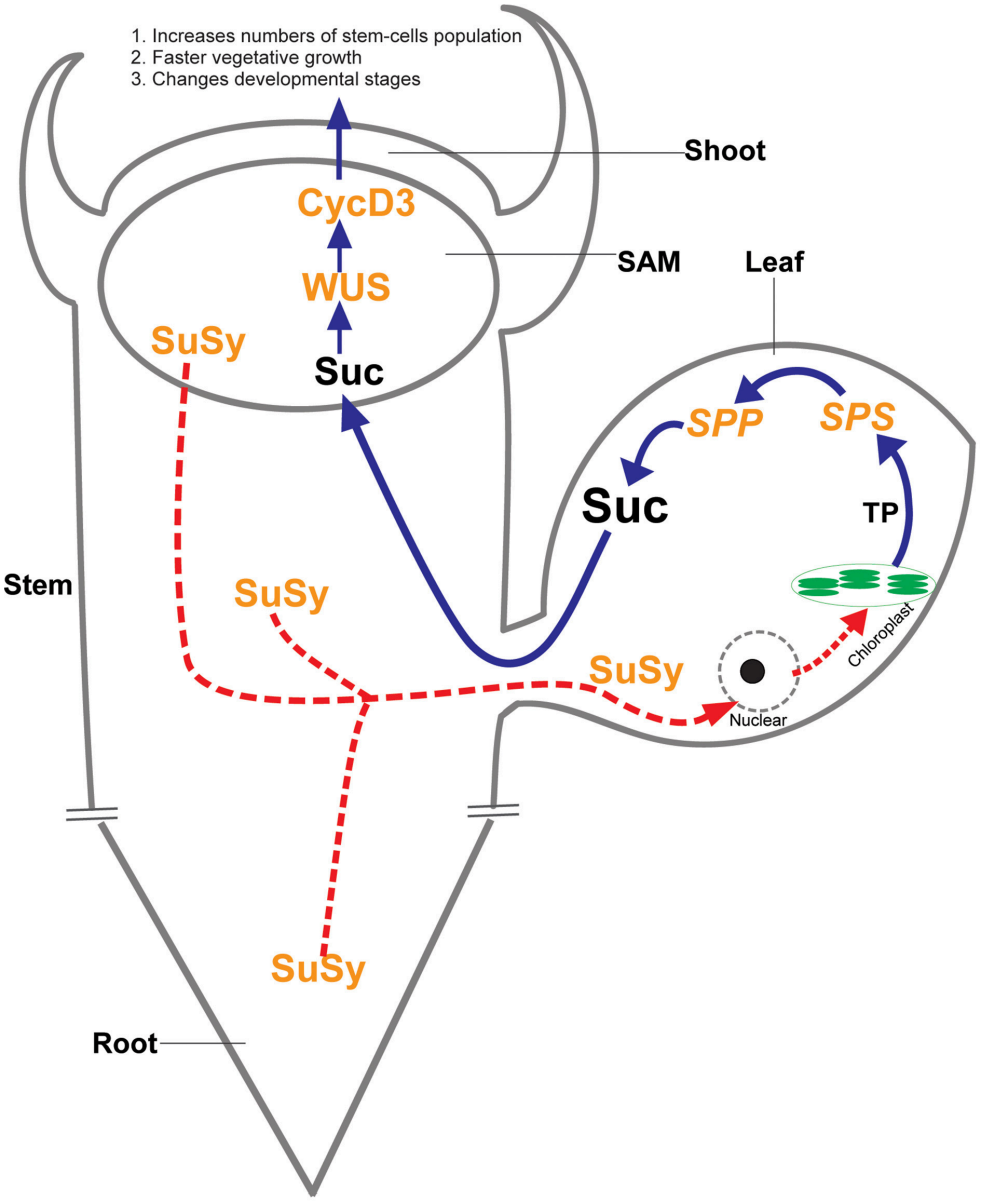
**Proposed unknown sucrose signaling pathway**. The abundant presence of SuSy protein in whole organs of the transgenic plants leads to unbalanced sucrose status, particularly in non-photosynthetic cells, may be the cause of an unknown sucrose signaling pathway that targeted to the nuclei of photosynthetic cells. Consequently, the nucleus increases photosynthetic efficiency in chloroplasts through an unknown mechanism and photosynthetic sucrose synthesis is upregulated so the plant can cope with sucrose demand in non-photosynthetic cells. Sucrose becomes abundant, which induces increased expression of the *WUSCHELL (WUS)* and *CycD3* genes that regulated shoot apical meristem (SAM) development. The increased development of SAM consists of an increase in the numbers of stem-cells, leading to faster vegetative growth and changes in developmental stages. This proposed pathway fits the pronounced phenotypic and morphological changes that occurred in the transgenic tobacco plants.

Photosynthetic and non-photosynthetic cells in plants communicate to convey nutrient status within the plant body; however, the nature of the nutrient signals and how such signals activate meristems to provide a sustained supply of new cells for plant growth is poorly understood. Our results strongly support the vital role of sucrose during plant growth and development. The more abundant presence of SuSy protein may be the cause of a novel sucrose signaling pathway, which, in turn, increases photosynthetic efficiency, and stimulates photosynthetic sucrose synthesis to allow the plants to cope with the sucrose demand. Our data help explain why heterologous SuSy activity leads to increased sucrose content and pronounced phenotypic and morphological changes in the transgenic tobacco plants (Figure 5).

## Conclusion

Sucrose is synthesized in photosynthetic cells and transported to non-photosynthetic cells and other regions to cope with energy demands during plant growth and development. Sucrose also acts as a signal to regulate its status between photosynthetic and non-photosynthetic cells. Besides the previously known sugar signaling mechanisms, which help plants change their development to adapt to energy conditions and the surrounding environment, we describe a novel sucrose signaling pathway that allows plants to respond to constant changes in sucrose status by regulating photosynthesis and photosynthetic sucrose synthesis. The existence of this signaling pathway is strengthened by the same phenomena during sugar metabolism, by chlorophyll, photosynthetic efficiency, and photosynthetic sucrose synthesis, and also by the pronounced phenotypic changes corresponding to increased sucrose in all six SuSy transgenic plants examined herein, which had a preference for sucrose-degrading SuSy activity. Future studies should focus on understanding the precise nature of the signaling and control mechanisms associated with increased plant sugar synthesis and biomass accumulation.

### Conflict of interest statement

The authors declare that the research was conducted in the absence of any commercial or financial relationships that could be construed as a potential conflict of interest.
