# Supplementary material for: Pronounced Phenotypic Changes in Transgenic Tobacco Plants Overexpressing Sucrose Synthase May Reveal a Novel Sugar Signaling Pathway
Source: Front Plant Sci. 2016 Jan 11;6:1216. doi: 10.3389/fpls.2015.01216 (PMC4707253; doi:10.3389/fpls.2015.01216)
Supplement: Supplementary file 1 [file Presentation1.PDF]

Supplementary Material

Pronounced phenotypic changes in transgenic tobacco plants overexpressing sucrose synthase may reveal a novel sugar signaling pathway

Quynh Anh Nguyen[1], **Sheng Luan**[2], **Seung Gon Wi**[3], **Hanhong Bae**[4], **Dae-Seok Lee**[3], and **Hyeun-Jong Bae**[1, 3, *]

*** Correspondence:** Prof. Hyeun-Jong Bae

Department of Bioenergy Science and Technology

Chonnam National University

Gwangju 500-757, Republic of Korea.

Tel: +82-62-530-2097. Fax: +82-62-530-0029.

E-mail address: baehj@chonnam.ac.kr (H.-J. Bae)

1. Supplementary Data

1.1. Supplementary Figures legends

Supplementary Figure S1. (**A**) Vector constructs for plants transformation. (**B**) Western blot confirmed the expression of *At.SuSy* in transgenic plants. Pattern profile of sucrose-degrading SuSy activity in the reaction with UDP (**C**) and without UDP (**D**). (**E**) Pattern profile of sucrose-synthesizing SuSy activity of wild-type (WT) and S1 to S6 transgenic plants. (**F**) Sucrose-degrading SuSy activity in different parts of transgenic and WT plants. (**G**) Sucrose-synthesizing SuSy activity in different parts of transgenic and WT plants. (**H**) Profile of TSS of S1 to S6 transgenic and WT plants. (**I**) Sucrose concentration (%) and TSS of leaves, stems, and roots of WT and S1 transgenic plants. (**J**) Starch content of leaves, stems, and roots of WT and S1 transgenic plants. (**K**) and (**L**) Profile of SPS and SPP enzymatic activity, respectively, of WT and S1 to S6 transgenic plants.

Supplementary Figure S2. (**A**) Sugar content in shoot tips of WT and S1 to S6 transgenic plants. (**B**) and (**C**) Profile of stem height and stem diameter, respectively, of WT and S1 to S6 transgenic plants from 30 to 120 DAG. (**D**) Rate of bifurcated stem in S1 to S6 transgenic plants.

Supplementary Figure S3. (**A**) Seedling stem cross-section stained with 1% toluidine blue (bar = 1 mm). (**B**) Horizontal section of seedlings apical, shoot stained with 1% toluidine blue (bar = 1 mm).

(**C**) Size of shoot apical area (height x width, mm^2) of WT and S1 transgenic seedlings in different light/dark time treatment experiment.

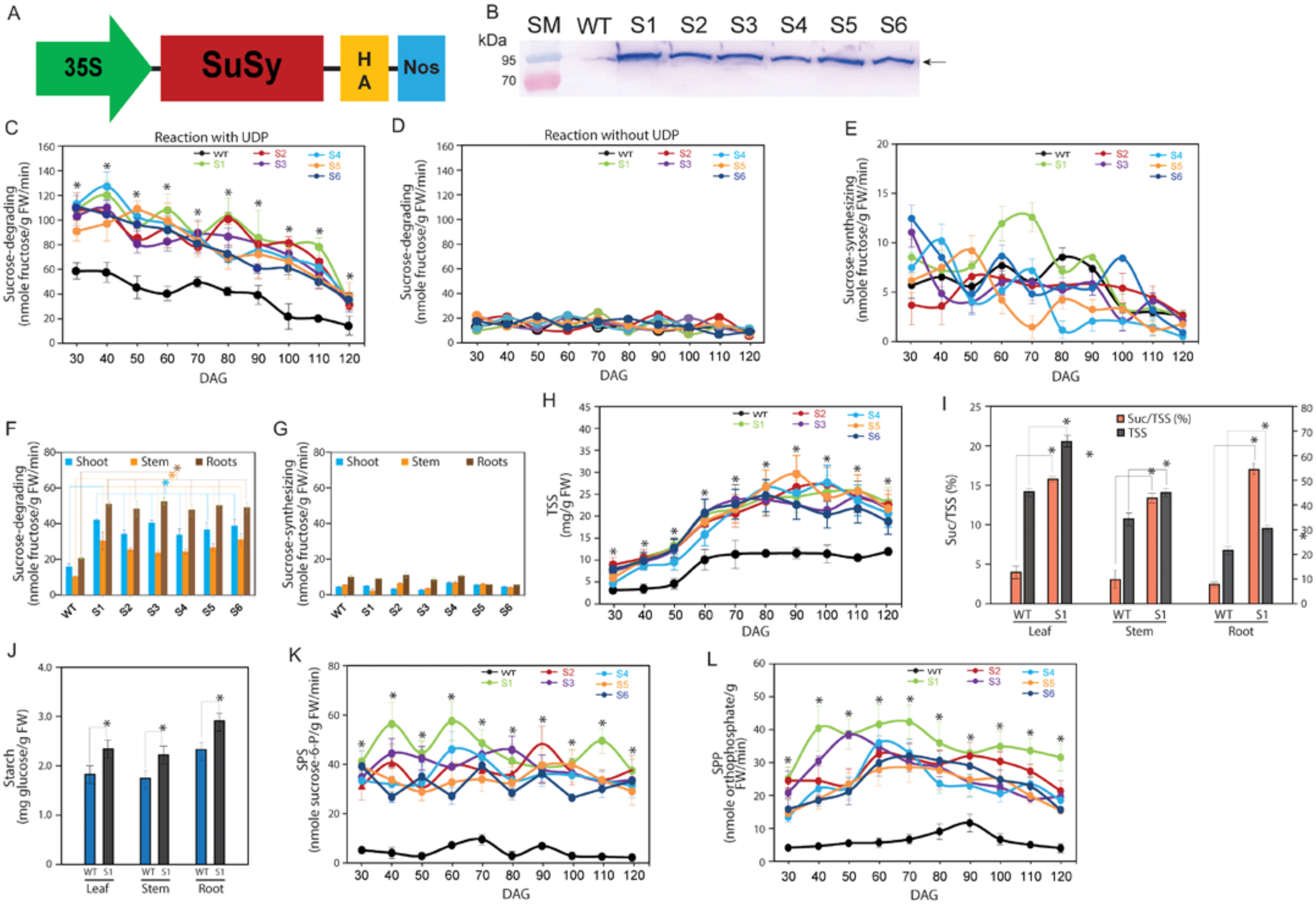

Supplementary Figure S1. (**A**) Vector constructs for plants transformation. (**B**) Western blot confirmed the expression of *At.SuSy* in transgenic plants. (**C**) and (**D**) Profile of sucrose-degrading SuSy activity and sucrose-synthesizing SuSy activity of wild-type (WT) and S1 to S6 transgenic plants. (**E**) Profile of TSS of S1 to S6 transgenic and WT plants. (**F**) Sucrose concentration (%) and TSS of leaves, stems, and roots of WT and S1 transgenic plants. (**G**) Starch content of leaves, stems, and roots of WT and S1 transgenic plants. (**H**) and (**I**) Profile of SPS and SPP enzymatic activity, respectively, of WT and S1 to S6 transgenic plants.

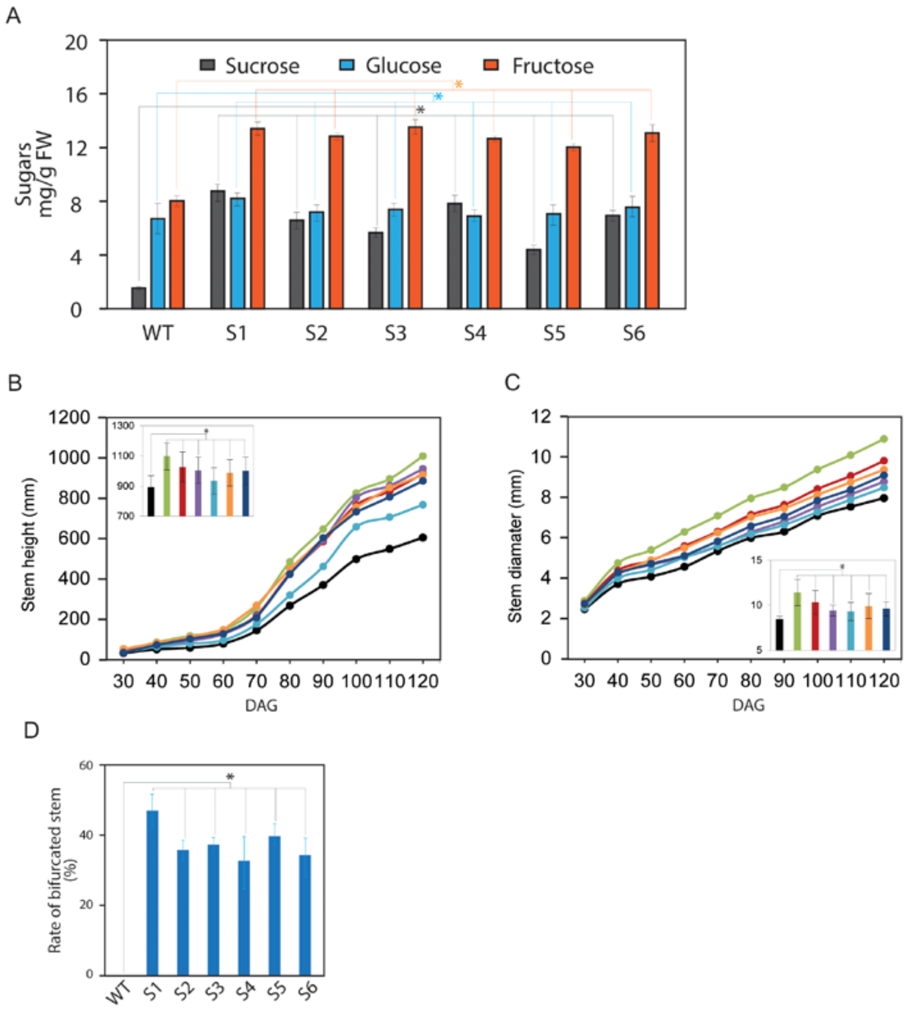

Supplementary Figure S2. (**A**) Sugar content in shoot tips of WT and S1 to S6 transgenic plants. (**B**) and (**C**) Profile of stem height and stem diameter, respectively, of WT and S1 to S6 transgenic plants from 30 to 120 DAG. (**D**) Rate of bifurcated stem in S1 to S6 transgenic plants.

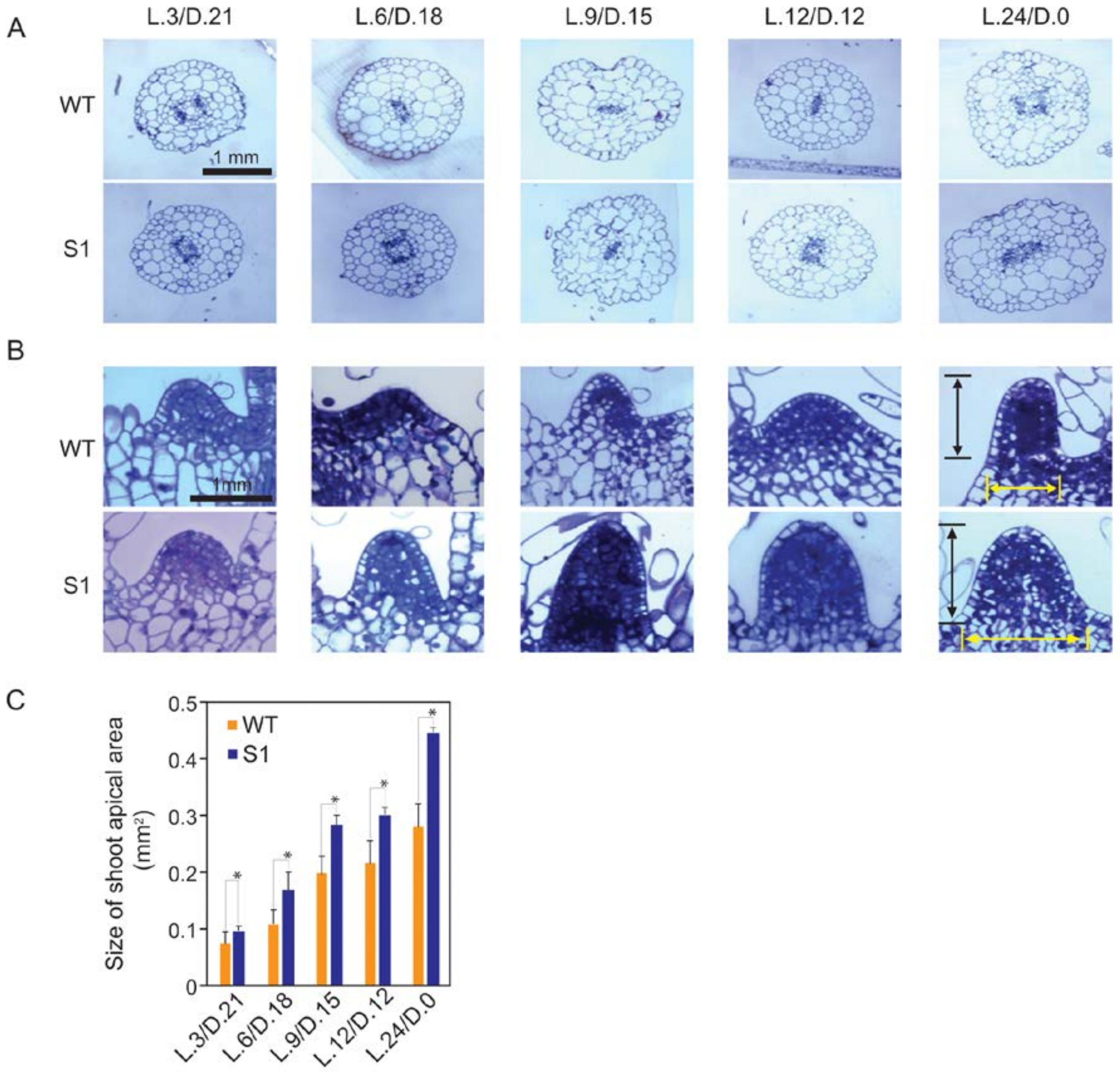

Supplementary Figure S3. (**A**) Seedling stem cross-section stained with 1% toluidine blue (bar = 1 mm). (**B**) Horizontal section of seedlings apical, shoot stained with 1% toluidine blue (bar = 1 mm). (**C**) Size of shoot apical area (height x width, mm^2) of WT and S1 transgenic seedlings in different light/dark time treatment experiment.

1.2. Supplementary Tables

Supplementary Table S1. List of used primers

No.	Gene name	Forward primer	Reverse primer
1	*At.SuSy1 (AT5G20830)*	5' ATG GCA AAC GCT GAA CGT ATG ATA ACG 3’	5' ATC ATC TTG TGC AAG AGG AAC 3'
2	*At.SuSy2 (AT5G49190)*	5’ ATG CCG ACT GGT AGG TTC GAG ACT ATG 3’	5' GTT CTC ATC TGT TGC CAG CGG 3'
3	*At.SuSy3 (AT4G02280)*	5’ ATG GCA AAC CCT AAG CTC ACT AGG GTT 3’	5' GTC ATC GGC GGT TGA AGG AAC 3'
4	*At.SuSy4 (AT3G43190)*	5’ ATG GCA AAC GCA GAA CGT GTA ATA ACG 3’	5' CTC TTC ATG AGC AAG AGG AAC 3'
5	*At.SuSy5 (AT5G37180)*	5’ ATG GAA ATG ACA TCT GGA TCG TTA GGC 3’	5' AGC ACC AAA CAA CCT GAA ACT 3'
6	*At.SuSy6 (AT1G73370)*	5’ ATG TCA TCT TCA TCT CAA GCT ATG CTT 3’	5' ATA CTC TTG AGC CGA GTT AGC 3'
7	*Nt.WUS (JQ686923.1)*	5' ATG CAC ATG AGA GGT GTT TG 3'	5' TTA AGG GGA ATT AGG AGA TC 3'
8	*Nt.CycD3.1 (AB243209.1)*	5' ATG GCA ATA GAA CAC AAT GAG CAA C 3'	5' TTA ATG AGG ACT ACC AAC AGC TTC 3'
9	*Nt.SPS (AF194022.1)*	5' GAA TTC ATG GCG GGA AAC GAT TGG AT 3'	5' TCT AGA ATA TAA GGC CAT AGC TGC TC 3'
10	*Nt.SPP (AY729655.1)*	5' GAA TTC ATG GAT CAG CTA ACC AGT GC 3'	5' TCT AGA GGA GAG GTA TTT GGT CCC AG 3'
11	*Nt.CHLG (FJ905101.1)*	5' ATG GCT TCT CTC CTC AAC TC 3'	5' TCA GTG GCT AGT TGC CAA AG 3'

Supplementary Table S2. Comparison of phenotypic characteristic of wild-type (WT) and S1 seedlings exposed to different light/dark time treatment.

	**L.3/D.21**		**L.6/D.18**		**L.9/D.15**		**L.12/D.12**		**L.24/D.0**	
	WT	S1	WT	S1	WT	S1	WT	S1	WT	S1
Number of leaf	3.8 ± 0.3	4.1 ± 0.3*	4.1 ± 0.4	4.8 ± 0.4*	4.3 ± 0.3	5.2 ± 0.4*	4.9 ± 0.3	5.5 ± 0.4*	5.2 ± 0.3	5.9 ± 0.5*
Size of leaf (mm^2)	8.2 ± 0.9	8.4 ± 0.8	10.5 ± 1.1	12.8 ± 0.9*	13.4 ± 1.6	23.7 ± 2.0*	15.3 ± 2.0	35.0 ± 3.2*	33.6 ± 3.1	51.0 ± 4.9*
Stem height (mm)	6.2 ± 0.6	6.3 ± 0.5	6.4 ± 0.6	7.4 ± 0.5*	8.3 ± 0.7	11.2 ± 0.8*	8.8 ± 0.7	13.2 ± 1.1*	10.4 ± 0.8	14.7 ± 1.7*
Fresh weight (mg/10 plants)	8.3 ± 0.8	8.5 ± 0.6	17.4 ± 1.5	20.4 ± 1.4*	26.5 ± 1.4	31.8 ± 2.1*	37.8 ± 2.9	49.6 ± 2.6*	50.4 ± 4.1	71.5 ± 5.4*

Means values were calculated from data obtained from individuals (n = 20) in each chosen transgenic line and WT. Asterisks (*) indicate significant differences determined by a Student's *t*-test (*$P < 0.05$).
